# Rare clinical image of chronic bilateral lymphatic filariasis with secondary bilateral hip osteonecrosis

**DOI:** 10.11604/pamj.2025.52.182.49425

**Published:** 2025-12-24

**Authors:** Sakshi Shankarrao Waghmare, Subrat Samal

**Affiliations:** 1 Department of Cardiovascular and Respiratory Physiotherapy, Ravi Nair Physiotherapy College, Datta Meghe Institute of Higher Education and Research, Sawangi (Meghe), Wardha, Maharashtra, India,; 2 Department of Musculoskeletal Physiotherapy, Ravi Nair Physiotherapy College, Datta Meghe Institute of Higher Education and Research, Sawangi (Meghe), Wardha, Maharashtra, India

**Keywords:** Osteonecrosis, avascular necrosis, lymphatic filariasis, total hip arthroplasty

## Image in medicine

Osteonecrosis of the femoral head (ONFH) is a progressive disorder caused by vascular compromise to subchondral bone, leading to joint collapse and disability. It is associated with multiple risk factors, but its coexistence with chronic lymphatic filariasis is rarely reported. Early diagnosis and management are crucial to prevent irreversible hip destruction. This case highlights the rare association of chronic lymphatic filariasis with bilateral femoral head osteonecrosis, the limited benefit of core decompression in advanced disease. A 52-year-old male, known case of bilateral lymphatic filariasis for forty years, presented with progressive dull aching pain in the right hip and difficulty in ambulation for one year. The pain was progressive, aggravated by weight-bearing and relieved by rest. Six months earlier, he had undergone core decompression with bone grafting, without symptomatic relief. Subsequently, symptoms worsened with functional limitation. Radiographs revealed bilateral avascular necrosis of femoral heads (R>L), and the patient underwent right total hip arthroplasty with physiotherapy rehabilitation.

**Figure 1:**
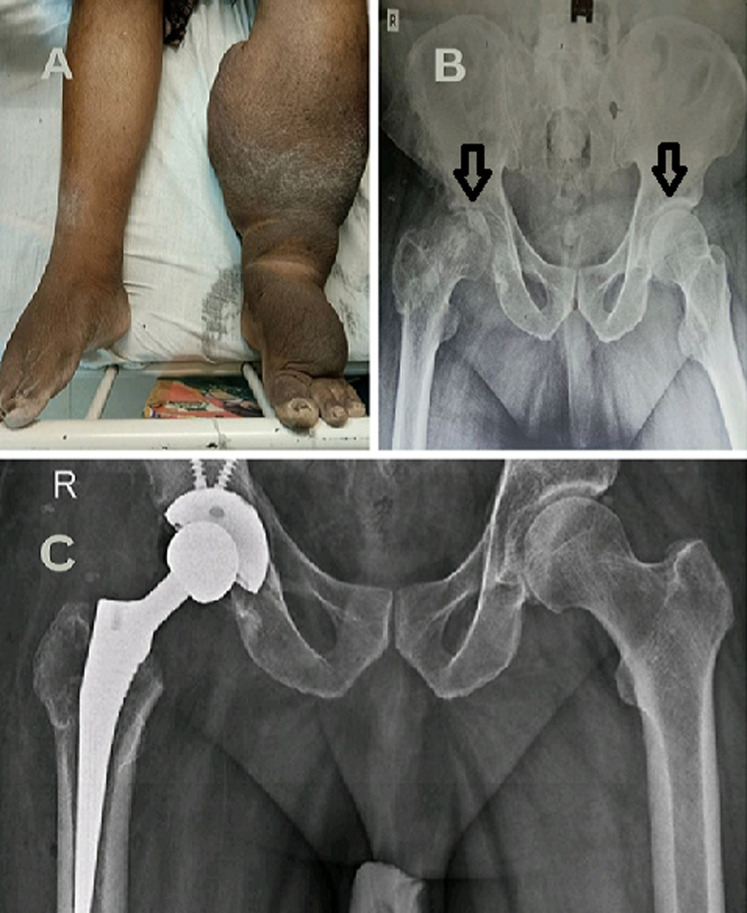
A) chronic lymphatic filariasis of bilateral lower limb (L>R); B) pre-operative Pelvic radiograph showing bilateral femoral head osteonecrosis (R>L); C) post-operative radiograph showing right total hip arthroplasty in situ

